# Inside the Dragon: non-invasive analysis of the trunks of the monocot *Dracaena draco* via electrical resistivity tomography

**DOI:** 10.3389/fpls.2026.1897843

**Published:** 2026-09-22

**Authors:** Adriano Losso, Vanesa Cristina Luis Díaz, Agueda María González-Rodríguez, Stefan Mayr

**Affiliations:** 1 Department of Botany, University of Innsbruck, Innsbruck, Austria; 2 Department of Botany, Ecology and Plant Physiology, Universidad de La Laguna, Canary Islands, La Laguna, Spain

**Keywords:** canopy die-back, dragon tree, electrical resistivity tomography, secondary growth, tree assessment

## Abstract

*Dracaena draco* is an evergreen monocotyledonous species endemic to the Macaronesian region and the Moroccan Anti-Atlas, and is endangered due to severe habitat fragmentation. Additionally, many *D. draco* specimens growing in public and private gardens have recently suffered substantial canopy damage, with some trees losing their entire canopy. We used minimally invasive electrical resistivity (ER) tomography to assess the trunks of *D. draco* individuals suffering different levels of canopy damage. The extent of canopy damage was used to categorize trees into three groups: visibly undamaged (0%), moderately damaged (25-55%) and severely damaged (70-95%). ER tomograms enabled novel insights into the trunks of *D. draco*, by displaying homogeneous and concentric patterns corresponding to primary (higher ER) and secondary growth (lower ER). A progressive and significant increase of ER was found with increasing percentage of canopy damage. Visibly undamaged trees exhibited significantly lower ER (<250 Ωm) than severely damaged trees (>250 Ωm), while moderately damaged trees had ER falling between the two extremes. This study demonstrates that ER tomography can be a useful tool for analyzing structural and functional traits also in the trunks of monocotyledonous trees having secondary growth. Results showed an imbalanced ER distribution and higher values in the trunk of trees with a severely damaged canopy. This indicates possible wood dehydration and/or decreased electrolyte content and variation in tissue density.

## Introduction

*Dracaena draco* (L.) L. subsp. *draco*, commonly known as the “Dragon Tree” due to its mythical association with dragons (when cut they exude a red resin referred to as dragon’s blood), is an evergreen monocotyledonous species endemic to the Macaronesian region (i.e., archipelagos of Madeira, Canary Islands, and Cape Verde) and the Moroccan Anti-Atlas, but also commonly found in many gardens around the world as an ornamental plant. *D. draco* has a tree-like growth habit and is characterized by some peculiar features such as regular rhythms of vegetative growth and flowering that occur at long time intervals of 10 to 20 years (Byström, 1960; Symon, 1974; Mägdefrau, 1975), which are very atypical in the plant kingdom. The growth pattern gives the mature tree an umbrella-like appearance, resulting in a well-organized canopy consisting of several orders of branches (Byström, 1960; Symon, 1974; Mägdefrau, 1975). D. draco is also one of the few monocots with secondary growth (e.g., Jura-Morawiec, 2017), which consists of a network of vascular bundles (secondary phloem and secondary xylem) that are formed by the activity of the monocot cambium and are embedded within the ground parenchyma (Carlquist, 2012; Jura-Morawiec, 2015; Jura-Morawiec et al., 2021) (see also **Supplementary Figure S1**). This added vascular tissue forms a distinctive ‘wood’, which is essential for providing mechanical support and has enabled some specimens to grow to a considerable size. A good example is the iconic “El Drago Milenario” tree monument on the island of Tenerife, which is 21 m tall, has a circumference of 20 m and is estimated to be over 400 years old.

In the case of the Canary Islands, this tree species was formerly widespread throughout the thermosclerophyllous woodlands (100–700 m a.s.l.; Fernández-Palacios et al., 2008; Maděra et al., 2020), whereas wild populations have dramatically decreased in the last century, with only few natural individuals remaining on some islands. Nowadays, D. draco specimens can mostly be found in private and public gardens across the island. Due to the restricted area of occupancy, severe habitat fragmentation and ongoing habitat quality decline (Silva et al., 2021), as well as a very low natural regeneration (González-Castro et al., 2019), the IUCN has assessed D. draco as an “Endangered” species. In Tenerife, small wild populations are considered relatively stable as most remaining individuals grow in inaccessible areas that protect them from significant threats. However, their survival can still be impacted by storms, grazing and invasive plants (Silva et al., 2021).

In recent years, substantial canopy damage has affected many D. draco specimens in public and private gardens, with some trees even losing their entire canopy. As the cause of these damages is unknown, and our understanding of this species’ water relations is still limited (e.g., Nadezhdina et al., 2015; Tulik et al., 2022), this study assessed D. draco individuals with varying degrees of canopy damage using electrical resistivity (ER) tomography. ER tomography is a minimally invasive technique that provides detailed insights into the distribution of water within the trunks of living trees without compromising their health (Daily et al., 2005), and has already been successfully used to assess the fitness of trees exposed to various stresses, including drought (Knüver et al., 2022; Oberleitner et al., 2022), fire (Bär et al., 2019b), wetwood (Martin et al., 2021), parasites (Park et al., 2025) and fungal infections (Bieker et al., 2010). It is based on the analysis of cross-sectional resistivity distributions, which are generated by creating an electrical field and subsequently measuring the electrical conductivity (and ER, as its reciprocal). Finally, ER tomograms can be used to visualize the distribution of resistivities across entire cross sections. Cross-sectional ER patterns are caused by the varying electrical properties of wood, which are primarily influenced by moisture content, electrolyte concentration, and wood density (Al Hagrey, 2007; Bieker and Rust, 2010; Bieker et al., 2010; Guyot et al., 2013; Bär et al., 2019a). Therefore, a low ER indicates higher levels of wood moisture and/or electrolytes, as well as lower wood density.

To our knowledge, ER tomography has not yet been used on monocots, but we expected this technique to also provide insights into the trunks of D. draco. As the method is minimally invasive, it should enable the assessment of cross-sectional patterns in the trunks without relevant impacts on the endangered trees. We hypothesized that (i) the water content of visibly undamaged D. draco is highest in the trunk periphery because this tissue is formed to supply the youngest and largest parts of the crown, and (ii) canopy damage corresponds to imbalanced water distribution in the trunk. Trees with the most severe canopy damage were expected to have overall higher ER, as a potential consequence of reduced trunk moisture and/or electrolytes content, and lower wood density.

## Material and methods

### Plant material

Measurements were performed on trunks of fifteen *Dracaena draco* (L.) L. subsp. *draco* trees growing in the campus of Universidad de La Laguna (La Laguna) and in Parque del Drago (Icod de los Vinos) (both in Tenerife, Canary Islands) in April 2024 and October 2025. Trees ranged between 2.5 to 8 m in height and 27 to 53 cm in trunk diameter (see **Table 1**) and were selected based on their different levels of canopy damage. The extent of canopy damage was assessed as a percentage of the full canopy, with 0% indicating no damage (complete leaf rosettes and green leaves) and 100% indicating total damage to the entire crown (no leaves on terminal branches). Trees were then categorized into three groups according to the extent of their canopy damage: visibly undamaged (0%; n = 7), moderately damaged (25-55%; n = 5) and severely damaged (70-95%; n = 3) (see **Figure 1**, **Table 1**). The assessment of the canopy damage was performed as a visual estimation directly in the field by all the authors (mean values of all individual assessments were used in further data analysis), and was based on the amount of terminal rosette loss from the total number of branches (see **Figure 1**). Selected trees did not have the characteristic aerial roots that are formed by younger trees in response to damages and/or under environmental stress (see Krawczyszyn and Krawczyszyn, 2014), were not artificially pruned, and showed no major visible fresh wounds, signs of decay, or symptoms of pathogen. All the trees were planted in garden areas, which protected them from damage and allowed them to be watered in case of drought.

**Table 1:** Tree number, percentage (%) and level of canopy damage, trunk diameter (cm), tree height (cm), minimum, maximum, mean (ER_mean_), central (ER_center_) and peripheral (ER_peripheral_) electrical resistivity (Ωm), as well as measuring site and date, of each measured *Dracaena draco* tree.

Tree n.	Canopy damage (%)	Level of damage	Trunk diameter (cm)	Tree height (cm)	Minimum ER (Ωm)	Maximum ER (Ωm)	ER_mean_ (Ωm)	ER_center_ (Ωm)	ER_periphery_ (Ωm)	Site	Measuring date
1	0	visibly undamaged	39.2	650	140	237	189.05 ± 0.81	208.32 ± 0.94	178.15 ± 2.28	Universidad de La Laguna	2-Apr-24
3	0	visibly undamaged	35.7	700	182	251	222.82 ± 0.55	235.77 ± 1.8	214.84 ± 1.59	Universidad de La Laguna	2-Apr-24
4	0	visibly undamaged	30.3	650	112	253	202.15 ± 1.49	231.61 ± 2.70	166.15 ± 2.24	Universidad de La Laguna	2-Apr-24
10	0	visibly undamaged	42.7	520	143.3	240.9	231.15 ± 1.52	254.94 ± 1.79	210.31 ± 4.07	Parque del Drago	28-Oct-25
11	0	visibly undamaged	33.1	500	104.1	221.5	192 ± 0.49	181.98 ± 0.15	183.29 ± 0.92	Parque del Drago	28-Oct-25
12	0	visibly undamaged	36.9	300	111.7	253.4	174 ± 0.74	188.33 ± 0.65	155.8 ± 1.27	Parque del Drago	28-Oct-25
5	0	visibly undamaged	27.1	500	135	317	169.2 ± 0.98	201.52 ± 2.13	144.73 ± 0.93	Universidad de La Laguna	2-Apr-24
15	25	moderately damaged	53.5	700	102.4	271.1	210.2 ± 0.99	262.97 ± 0.99	194.45 ± 1.88	Parque del Drago	28-Oct-25
14	30	moderately damaged	40.8	468	136.6	315.3	196.6 ± 1.41	313.89 ± 0.47	165.48 ± 1.18	Parque del Drago	28-Oct-25
13	45	moderately damaged	38.2	800	124.6	399.5	232.9 ± 1.99	364.22 ± 5.98	185.62 ± 1.26	Parque del Drago	28-Oct-25
6	50	moderately damaged	31.8		100	278	192.31 ± 1.67	249.41 ± 3.64	149.91 ± 2.83	Universidad de La Laguna	2-Apr-24
17	55	moderately damaged	32.8	550	70.8	483.9	207.7 ± 1.99	249.85 ± 3.63	188.85 ± 5.37	Parque del Drago	28-Oct-25
16	70	severely damaged	29.9	300	53.3	778.9	231.2 ± 2.98	368.59 ± 10.89	212.68 ± 6.89	Parque del Drago	28-Oct-25
7	80	severely damaged	35	250	174	487	283.89 ± 2.70	222.7 ± 5.16	309.38 ± 6.61	Universidad de La Laguna	2-Apr-24
2	95	severely damaged	28.7	500	246	364	304.07 ± 1.16	372.1 ± 1.17	272.54 ± 1.61	Universidad de La Laguna	2-Apr-24

**Figure 1:**
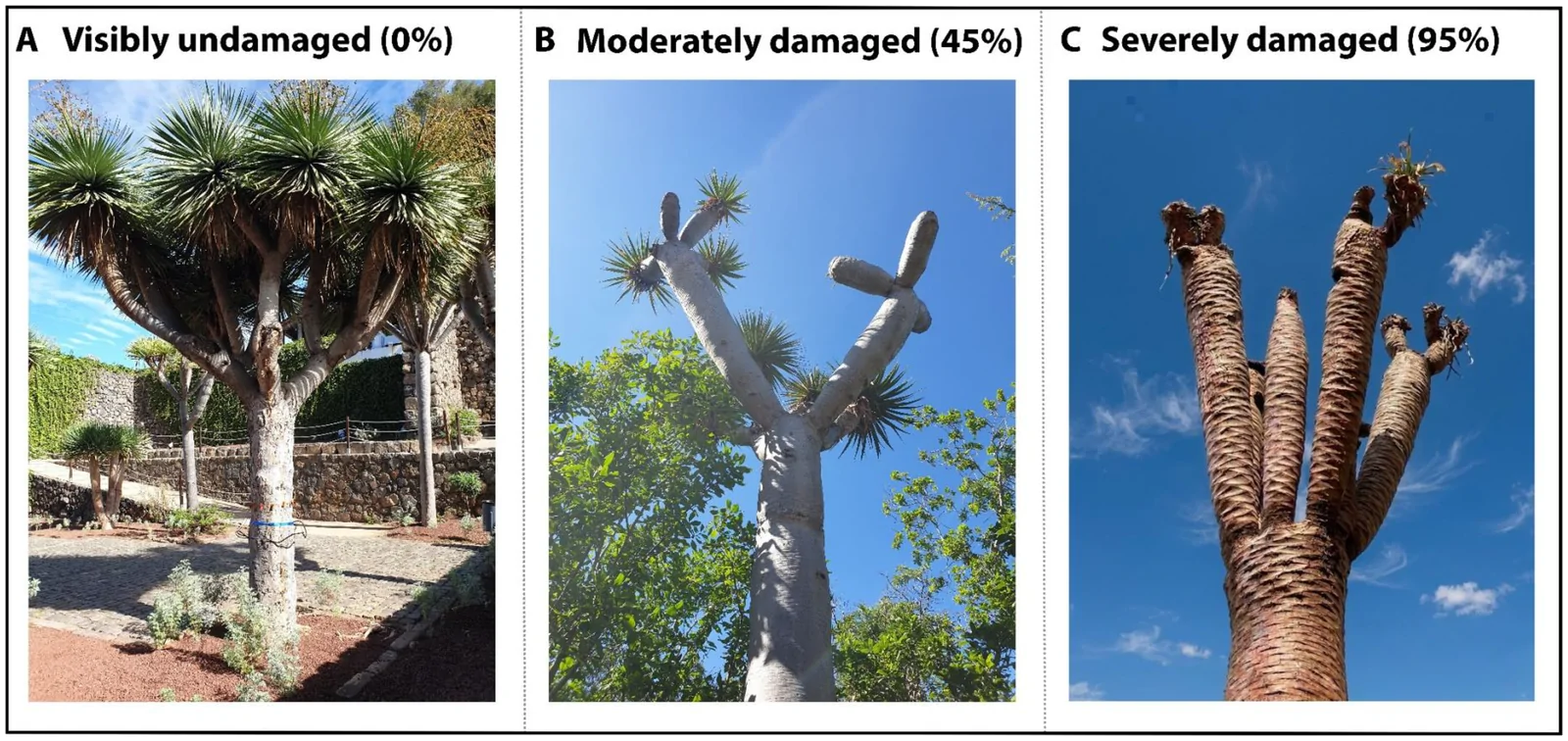
Representative photos for *Dracaena draco* trees with different levels of canopy damage: visibly undamaged (with 0% damage) **(A)**, moderately damaged (45% of damage) **(B)** and severely damaged (95% of damage) **(C)**.

### Electrical resistivity tomography

Electrical resistivity (ER) tomography enables the *in vivo* imaging of cross-sections of tree trunks *in situ* and was used in our study to analyze the trunks of visibly undamaged, moderately damaged, and severely damaged *D. draco* trees. This allows the visualization of patterns of moisture, density, and ion content in the trunk tissues. For ER measurements, we used a multichannel resistivity system (PiCUS: TreeTronic, Argus Electronic GmbH). The system was attached to each tree and connected via electrodes to 12 stainless steel nails (length 5 cm), which were installed at a constant height (120 ± 20 cm, depending on the surrounding terrain) above the ground and distributed equally around the trunk (see **Supplementary Figure S2**). The nails were sterilized with 70% ethanol prior to measurements and inserted 5–8 mm into the trunk counterclockwise, starting with a northward orientation, until firm contact with the tissues below the periderm was established. Due to the small and homogeneously round shape of the trunk, a circular cross-section was assumed. The temperature remained constant throughout the measurements, averaging 22 ± 2 °C.

Electrical voltages were applied to all installed electrodes (pairwise, all combinations), and the electrical field data were processed using inversion algorithms (for details, see Günther et al., 2006; Rücker et al., 2006) in the PiCUS software (PiCUS TreeTronic Q74, Argus Electronics GmbH, Germany). For a better, more comparable resolution, the smoothness and the mesh resolution were always set to 20 and 7, respectively. The cross-sectional distribution of the ER was then calculated using a circular tree geometry. The position, size and ER value of each triangle area within the tomogram, as determined by the measurement lines between the installed electrodes, were then exported and visualized using a color scale ranging from blue (low ER) to red (high ER), via green and yellow. As the area of a triangle varies depending on its radial position, the weighted ER (ER_w_; Ωm) was calculated for each triangle as:


$$ER_{w}=(ER*A)/\;A_{mean}$$

where A (cm^2^) is the area of an individual triangle and A_mean_ (cm^2^) is the mean area of all triangles. The average ER of each entire cross-section was then calculated as the mean ER_w_ of all the triangles. In addition, ER_center_ and ER_peripheral_ were calculated as the mean of ER_W_ of triangles within 0-10% and 90-95% relative radial position, respectively (Ganthaler et al., 2019). These areas were defined in order to capture representative values for innermost and peripheral regions and to exclude local ER variations around electrodes. In addition, ER_w_ was used to calculate the average ER for each total cross-section and for ten radially consecutive concentric segments. The segments were defined by their relative position from the center (0-10%) to the outermost peripheral part of the trunk (90-100%) to account for differences in absolute trunk diameter. This procedure allowed better comparison between the three groups (visibly undamaged, moderately damaged, and severely damaged).

### Statistics

To analyze the relationship between canopy damage (expressed as a percentage) and ER parameters (i.e., maximum, minimum, mean, central and peripheral) for each individual tree, a linear regression was fitted using the ggplot2 R package (Wickham, 2016) and the linear model fitting function (lm). Differences between ER_w_, ER_center_, and ER_peripheral_ of visibly undamaged, moderately damaged, and severely damaged trees were tested using a one-way ANOVA followed by Tukey’s *post hoc* comparison, after checking normality and homogeneity with Shapiro-Wilk’s and Levene’s tests, respectively. We implemented linear mixed-effects models using the lmer function in the lme4 R package to describe the relationship between the ER of different relative positions within the trunk diameter and canopy damage, site/date, tree diameter and height (Bates et al., 2015). To account for repeated measurements (pseudoreplication), we included tree identity as a random effect to satisfy the assumptions of a linear model. The formula for the mixed-effects model was: ER ~ canopy damage × radial position + site/date + diameter + height + random effect of tree identity. For moderately damaged tree n. 6 (see **Table 1**), the model did not take its tree height into account, as the tree had been cut down before height measurements could be taken. ANOVA and Akaike information criterion (AIC) were used to identify the model with the best fit. Pearson and Cramer’s V correlation analyses were used to test the relationship between the parameters used in the model. A linear regression was fitted using the ggplot2 R package (Wickham, 2016) and the linear model fitting function (lm) to further analyze the relationship between tree height and ER parameters (i.e., maximum, minimum, mean, central and peripheral) for each individual tree. All statistical data were analyzed with R version 4.5.1 (R Core Team, 2025) at a probability level of 5%.

## Results

### ER tomograms

ER tomograms of visibly undamaged *D. draco* displayed homogeneous and concentric patterns (**Figure 2**). In particular, a clear distinction could be observed between the outermost peripheral layers and the innermost part of the trunk, which were characterized by overall lower (< ca. 160 Ωm; blues in **Figure 2**) and higher ER values (> ca. 170 Ωm; greens/yellows in **Figure 2**), respectively. These homogeneous, concentric patterns and ER distribution were maintained at each level of canopy damage (**Supplementary Figure S3**). However, overall ER values increased as canopy damage increased (**Supplementary Figure S3**).

**Figure 2:**
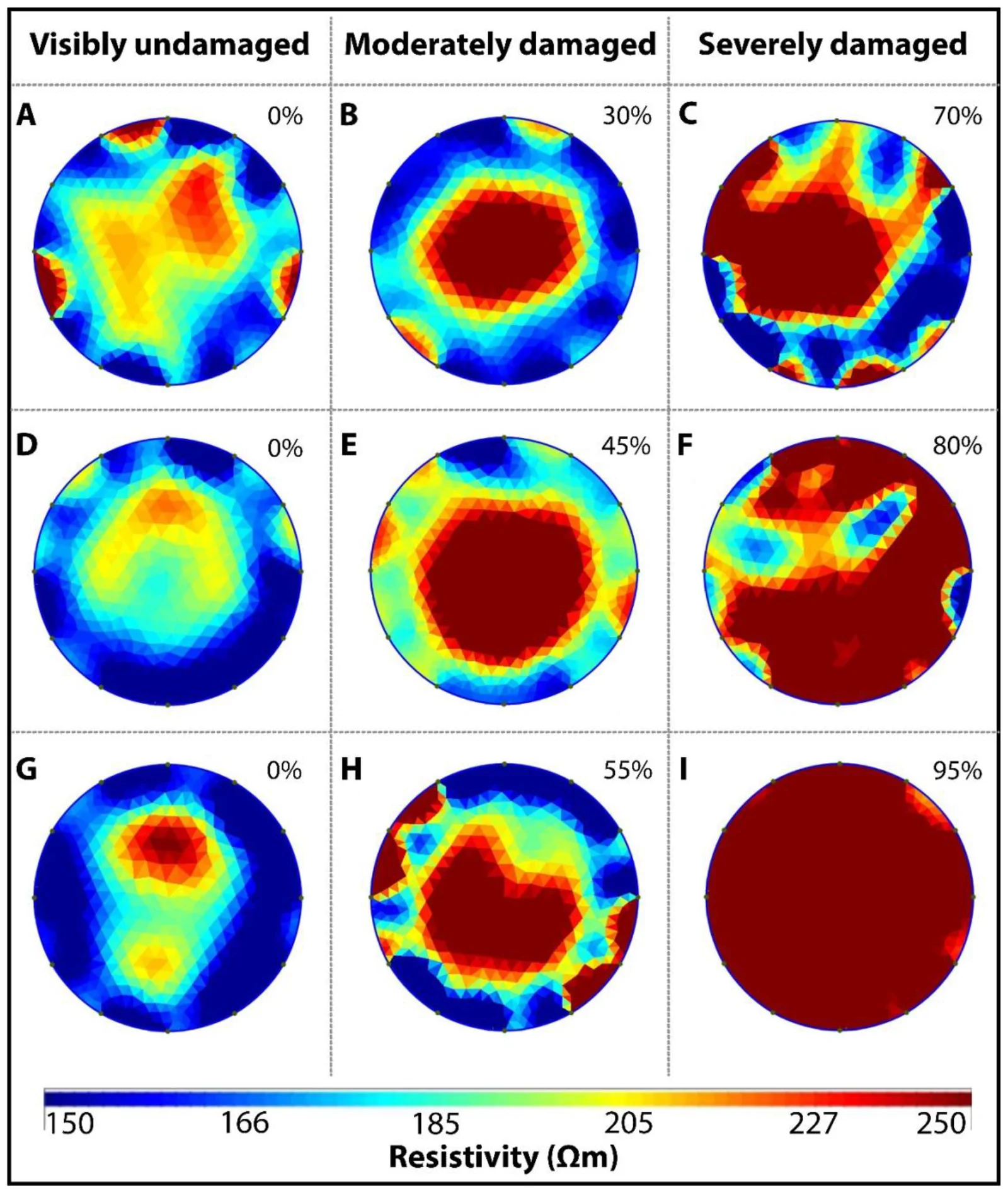
Electrical resistivity tomograms of three *Dracaena draco* trees per canopy damage level: visibly undamaged (with 0% damage; **(A, D, G)**; n = 7), moderately damaged (25-55% of damage; **(B, E, H)**; n = 5) and severely damaged (70-95% of damage; **(C, F, I)**; n = 3) (see also **Figure 1**). The corresponding level of canopy damage is given on the right top of each tomogram. In tomograms, areas of low resistivity are indicated by blue color while areas of high resistivity are indicated by red color. Note that the limit of the displayed resistivity ranges was set manually to optimize visualization, and ER_min_ and ER_max_ values may exceed these limits.

### ER_mean_, ER_center_, and ER_peripheral_

ER in the trunk cross-sections of *D. draco* ranged between 53.3 and 778.9 Ωm, with highest ER values found on a specimen with severe canopy damage (**Table 1**). The percentage of canopy damage was found to be significantly related to maximum ER (p < 0.01), ER_mean_ (p < 0.01), ER_center_ (p < 0.01), and ER_peripheral_ (p = 0.01) (**Figure 3**). ER_mean_ (p < 0.01), ER_center_ (p = 0.015), and ER_peripheral_ (p < 0.01) (**Figure 4**, **Table 1**) also differed between levels of canopy damage, and revealed lowest resistivities in the outermost peripheral part of the trunk and an increase in ER towards the center. In particular, ER of trees with severe damage on their canopy was always significantly higher (ER_mean_ 273.0 Ωm 95% CI 203.5 and 315.6 Ωm, p < 0.01; ER_center_ 321.1 Ωm 95% CI 224.6 and 417.6 Ωm, p < 0.02; ER_peripheral_ 264.9 Ωm 95% CI 209.6 and 320.1 Ωm, p < 0.01) than that of visibly undamaged trees (ER_mean_ 197.2 Ωm 95% CI 180.0 and 214.4 Ωm, ER_center_ 214.6 Ωm 95% CI 194.7 and 234.5 Ωm, ER_peripheral_ 179.0 Ωm 95% CI 159.5 and 198.5 Ωm). Trees with moderate damage to their canopy showed similar ER_mean_ (207.9 Ωm 95% CI 194.1 and 221.8 Ωm) and ER_peripheral_ (176.9 Ωm 95% CI 160.5 and 193.2 Ωm) to those of visibly undamaged trees and ER_center_ (288.1 Ωm 95% CI 244.1 and 332.0 Ωm) falling within those of visibly undamaged and severely damaged trees.

**Figure 3:**
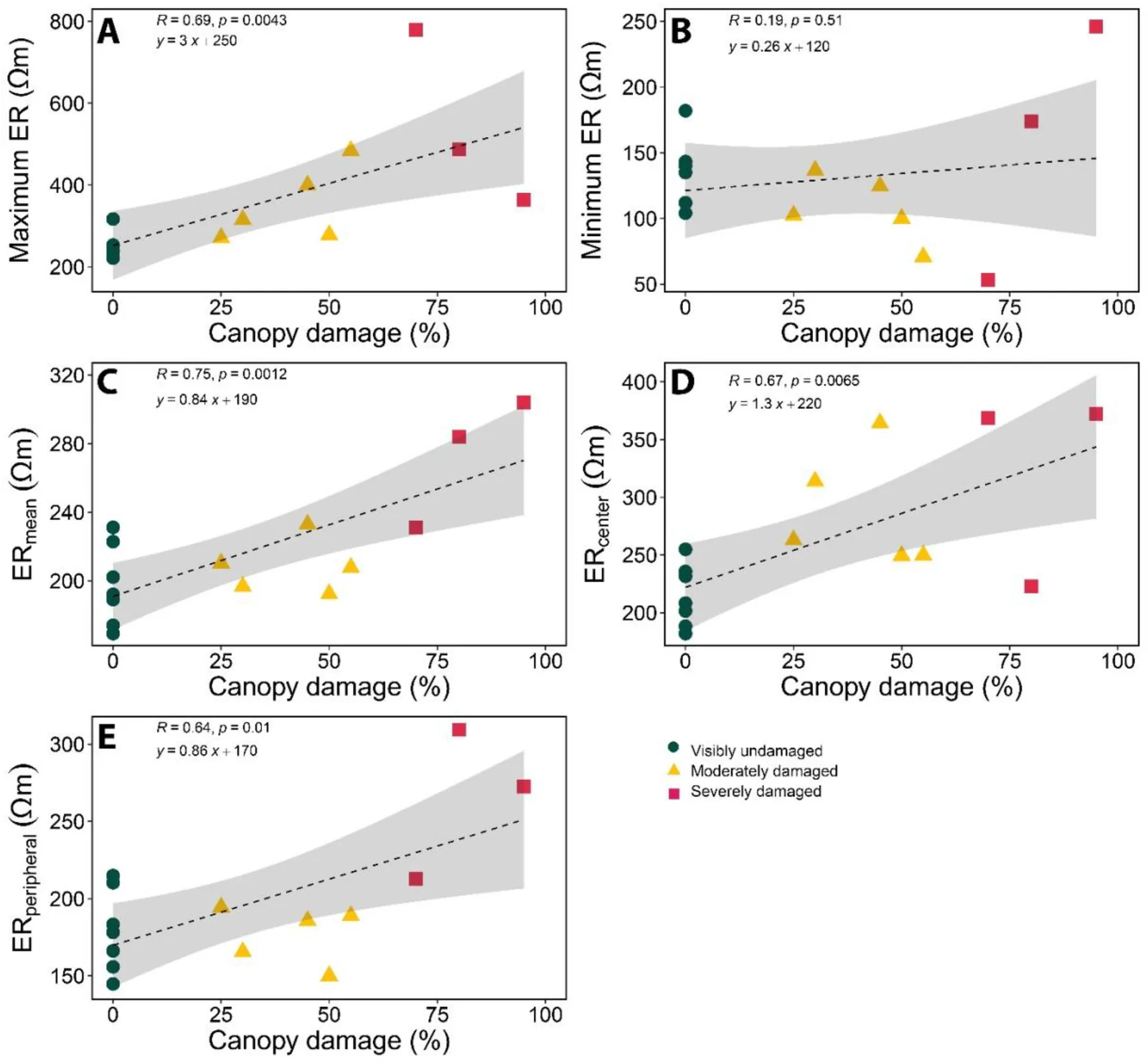
Percentage of canopy damage (%) *versus* maximum **(A)**, minimum **(B)**, mean **(C)**, central **(D)** and peripheral **(E)** electrical resistivity (Ωm) of *Dracaena draco* trunks of trees. Different colors and symbols indicate different damage classes: visibly undamaged (0%; green circles; n = 7), moderately damaged (25-55%; yellow triangles; n = 5) and severely damaged (70-95%; red squares; n = 3) (see also **Figure 1**). The dashed line shows the linear regression, and the gray shaded area represents its 95% confidence interval.

**Figure 4:**
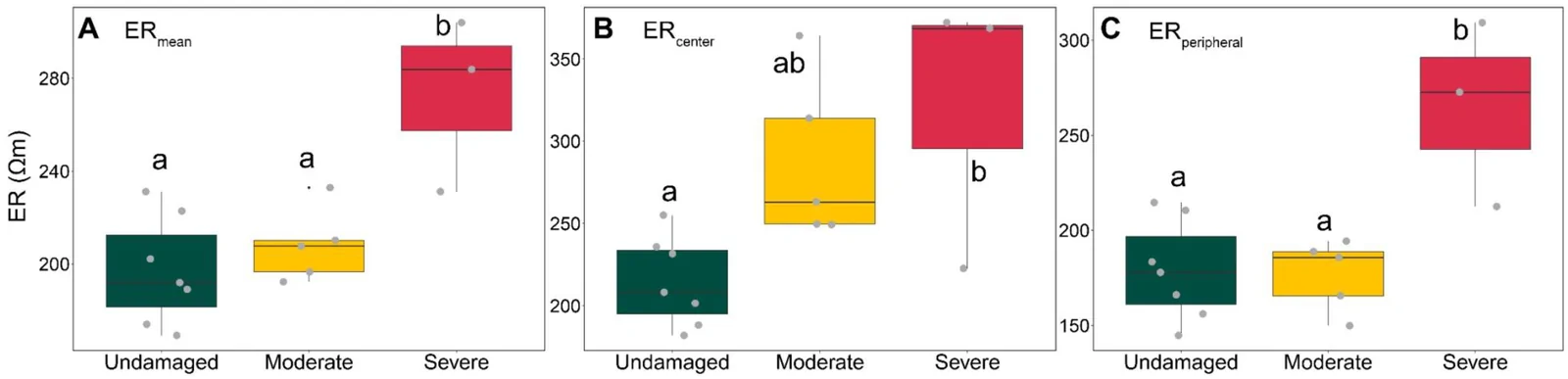
Mean (ER_mean_) **(A)**, central (ER_center_) **(B)** and peripheral (ER_peripheral_) **(C)** electrical resistivity (Ωm) of *Dracaena draco* trunks of trees with different levels of canopy damage: visibly undamaged (with 0% damage; green; n = 7), moderately damaged (25-55% of damage; yellow; n = 5) and severely damaged (70-95% of damage; red; n = 3) (see also **Figure 1**). Different letters indicate significant differences (p < 0.05) between different levels of canopy damage. Box center line represents median, box limits the upper and lower quartiles, whiskers the 1.5x interquartile range, while separated points represents outliers. Gray points represent individual tree-level values.

Tree height was not related to any ER parameter: maximum (p = 0.11) and minimum ER (p = 0.82), ER_mean_ (p = 0.6), ER_center_ (p = 0.74), and ER_peripheral_ (p = 0.27) (**Supplementary Figure S4**).

### ER profiles

The average radial distribution of absolute ER in the trunk sections of *D. draco* trees mirrored the same trend observed in ER_mean_, ER_center_, and ER_peripheral_, showing a consistent pattern across different levels of canopy damage, with higher absolute ER in the central region of the trunk and lower values in the outermost peripheral part (**Figure 5**). Trees with severely damaged canopies consistently exhibited higher absolute ER (> ca. 250 Ωm) at each relative position within the trunk than the visibly undamaged trees (**Figure 5**). Instead, trees with moderately damaged canopies exhibited high absolute ER at the center of the trunk (ca. 280 Ωm), comparable with that of severely damaged trees (**Figure 5**). Absolute ER then decreased progressively towards the outermost peripheral part of the trunk, reaching values similar to those of the visibly undamaged trees (ca. 170 Ωm).

**Figure 5:**
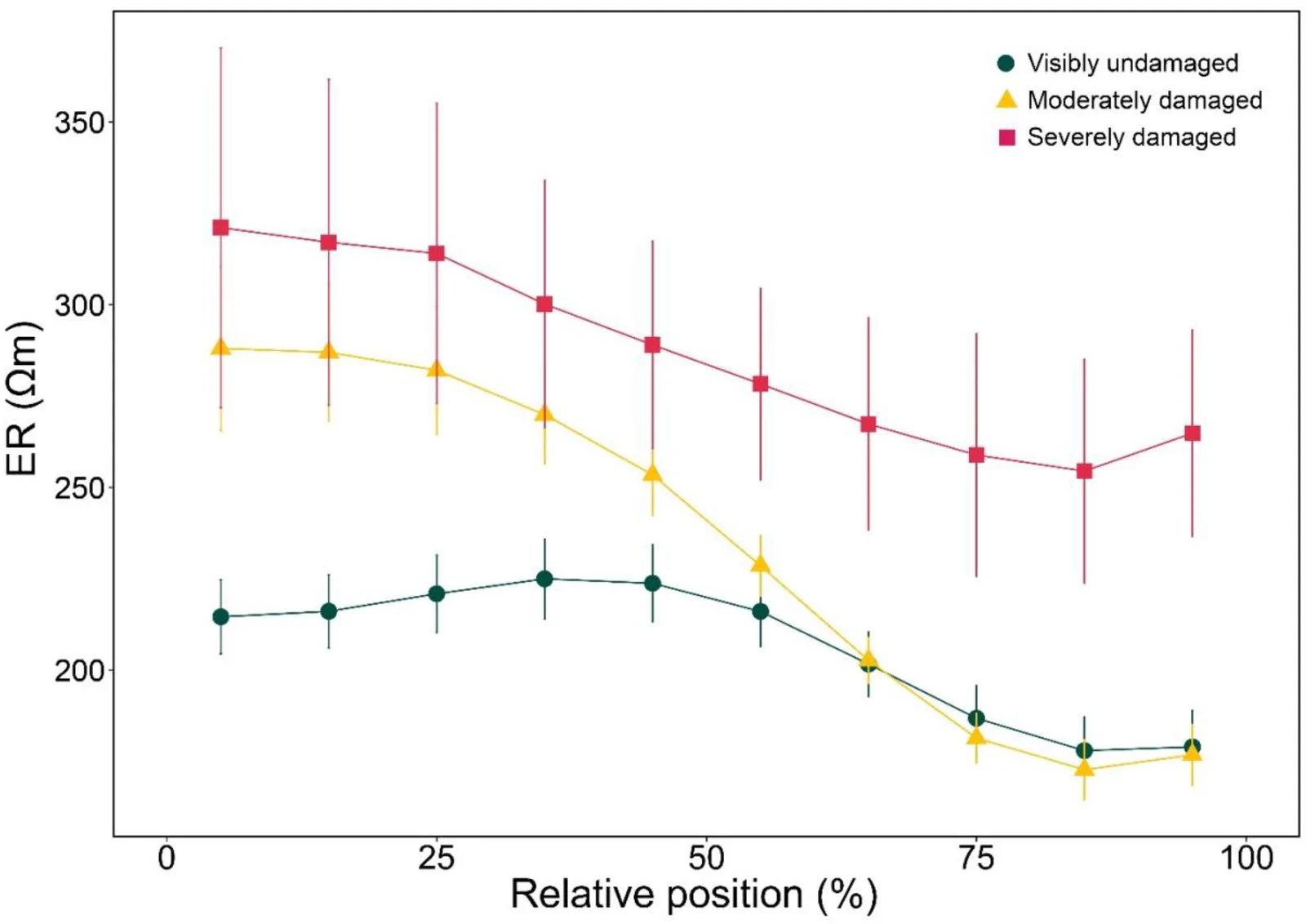
Radial distribution of electrical resistivity (ER; Ωm) *versus* relative trunk positions (%) of *Dracaena draco* trees with different levels of canopy damage: visibly undamaged (0%; green circles; n = 7), moderately damaged (25-55%; yellow triangles; n = 5) and severely damaged (70-95%; red squares; n = 3) (see also **Figure 1**). For each damage class, ER values were averaged per 10% increment and plotted against their relative position (5% = trunk center, 95% = outermost peripheral part of the trunk) to account for differences in absolute trunk diameter. Different letters indicate significant differences (p < 0.05) between different levels of canopy damage at a specific relative position. Mean ± SE.

The best linear mixed-effects model following AIC comparison was the one including canopy damage, relative trunk positions, site/date, tree diameter and tree height (AIC = 1263.60). Significant correlations were found between ER and both moderately (p < 0.001) and severely damaged trees (p < 0.001) as well as with tree height (p < 0.001). For ER, canopy damage, relative trunk positions, site/date, tree diameter and tree height, Pearson and Cramer’s V correlation coefficients were always lower than 0.5 except between tree height and damage, which was 0.58.

## Discussion

In this study, ER tomography enabled novel *in vivo* insights into the internal ER patterns of trunks of *D. draco* trees that had suffered varying degrees of canopy damage. As hypothesized, we observed pronounced differences in tomograms patterns and a progressive increase of ER with increasing canopy damage. ER of visibly undamaged trees (0% canopy damage) was significantly lower than that of severely damaged trees (70-95% canopy damage), while moderately damaged trees had values falling in between the two extremes. Results suggest an overall imbalanced ER distribution in the trunks of severely damaged trees.

This is the first study to use ER tomography on a monocotyledonous tree species, and tomograms of the visibly undamaged *D. draco* trees (i.e., not showing any canopy damage) displayed homogeneous and concentric patterns similar to those previously reported for conifers (Bieker and Rust, 2010; Guyot et al., 2013; Bär et al., 2019a; Ganthaler et al., 2019; Losso et al., 2020). Whereas in conifers, the tomograms clearly distinguish between heartwood (high ER) and sapwood (low ER) areas, in *D. draco*, differences in colors may indicate differences between areas of primary and secondary growth (**Figures 2A, D, G**, **Supplementary Figure S1**). Similarly, the ER values that probably correspond to the primary growth areas were higher than those of secondary growth areas, suggesting that the latter have a higher moisture higher electrolyte content and/or lower wood density (Just and Jacobs, 1998; Bieker and Rust, 2010; Guyot et al., 2013; Bär et al., 2019a; Losso et al., 2020). Based on anatomical measurements, Tulik et al. (2022) showed that *D. draco* appears to have adapted to withstand water deficits by spatially separating the xylem elements fulfilling supportive functions (i.e., lignified secondary growth) and from those with conductive functions (primary growth). However, the non-lignified primary parenchyma can deteriorate over time, thus resulting in the formation of air-filled areas in the central part of the trunk (as observed in very old specimens with hollow centers; see also **Supplementary Figure S1**) and likely increasing the overall ER. Such deterioration in basal and older sections of the stem could be compensated for by an axial widening, with conduit size increasing progressively from the tip to the base, as has been observed in other monocots (Petit et al., 2014). It would be interesting for further studies to make a direct comparison between ER and anatomical measurements to confirm the potential link between primary and secondary growth and changes in ER.

Despite the similarities in tomogram patterns with conifers, the ER_mean_ of visibly undamaged *D. draco* was substantially lower than that reported in previous studies on conifers (<500 Ωm; Bär et al., 2019a; Losso et al., 2020; Park et al., 2025) and was instead similar to that of healthy dicotyledonous angiosperm trees (Gora and Yanoviak, 2015; Bär et al., 2019a; Ganthaler et al., 2019; Olaoye et al., 2025). Dicotyledonous angiosperms tend to have a lower ER because of the larger proportion of living parenchyma, which contains more electrolytes than gymnosperm wood (Meerts, 2002; Bär et al., 2019a). The same could be assumed for *D. draco*, given the dense presence of ground parenchyma in both primary and secondary growth areas, where it embeds the so-called amphivasal arrangement (i.e., phloem surrounded by xylem; e.g., Jura-Morawiec and Wiland-Szymańska, 2014). Parenchyma cells have indeed been reported to be a main source of water and soluble carbohydrates in other *Dracaena* spp (Jura-Morawiec and Wiland-Szymańska, 2014; Jupa et al., 2017).

The extent of canopy damage in the *D. draco* trees under study was clearly reflected by tomograms (**Figure 2**) and respective ER values (**Figures 3**, **4**, **5**). A progressive and significant increase in maximum ER, ER_mean_, ER_center_, and ER_peripheral_ was found as the percentage of canopy damage increased (**Figure 3**). Overall, the visibly undamaged trees always had lower ER values (< ca. 250 Ωm), with moderately damaged trees showing transitional values before severely damaged trees reached the highest ER values (> ca. 250 Ωm and up to a max of 778.9 Ωm; see **Table 1**). It should be noted that only three individuals with severely damaged canopies were analyzed, and greater variation in trunk ER distribution may occur at this level of damage if more individuals were to be analyzed. These results may indicate a progressive dehydration of the whole trunk, and/or electrolyte depletions and changes in tissue status (i.e., increased density) with increasing degree of canopy damage. Such trend is similar to what has been previously observed in the trunk of both angiosperm and conifer trees exposed to drought stress (Ganthaler et al., 2019; Oberleitner et al., 2022), with highest ER values measured in severely stressed *D. draco* being similar to those of drought-stressed angiosperms (Ganthaler et al., 2019). However, it is unclear whether higher ERs are a consequence or a cause of the canopy damage observed in the trees under study. Further investigation into the relationship between water and electrolyte content and wood density along trunk profiles would provide a clearer explanation of the observed patterns.

Overall, tomograms always showed the same pattern as described above (i.e., decrease in ER between primary and secondary growth areas) at every level of canopy damage (**Figures 2**, **Supplementary Figure S3**), but had overall higher ER values with increasing damage (**Figure 4**). The radial distribution of absolute ER also supports these findings, indicating higher ER values in every relative trunk position of severely damaged trees compared to the visibly undamaged ones (**Figure 5**). Interestingly, moderately damaged trees showed higher absolute ER values in the central part of the trunk than the visibly undamaged trees, but the peripheral parts of both groups had the same ER values (**Figure 5**). This may indicate a different rate of damage-related changes between primary and secondary growth areas. Such differences may be due to the anatomical nature of the primary growth, partly because of the potential presence of air-filled areas resulting from the deterioration of non-lignified parenchyma, and partly because conduits in the primary growth tend to have thinner cell walls and wider conduits than those in the secondary growth (Tulik et al., 2022), most likely making them more prone to embolism under drought stress. Both cases would be responsible for an increase in ER. On the other hand, although the secondary conduits mainly provide mechanical support due to their twisted and intertwined structures, and thicker cell walls (Tulik et al., 2022), they may be able to retain more water when the plant is under stress. However, this difference gradually decreases as the canopy damage and ER values increase (**Figures 3**, **4**).

The present study indicates that ER tomography is a useful tool for analyzing structural and functional traits also in the trunks of monocotyledonous trees having secondary growth. Moreover, the results confirm the suitability of this method for minimally invasive investigations of tree trunks, providing relevant insights for evaluating the stress levels to which they are subjected. Further research is recommended on more monocotyledonous species, both with and without secondary growth, alongside measurements of relevant tissue parameters (e.g. density, moisture and electrolyte content), in order to gain a broader understanding of ER variation among monocotyledonous species.

## Data Availability

The raw data supporting the conclusions of this article will be made available by the authors, without undue reservation.
