# Supplementary material for: Inside the Dragon: non-invasive analysis of the trunks of the monocot Dracaena draco via electrical resistivity tomography
Source: Front Plant Sci. 2026 Sep 22;17:1897843. doi: 10.3389/fpls.2026.1897843 (PMC13638648; doi:10.3389/fpls.2026.1897843)

Figure S1. A cut trunk of *Dracaena draco* with visible primary and secondary growth.

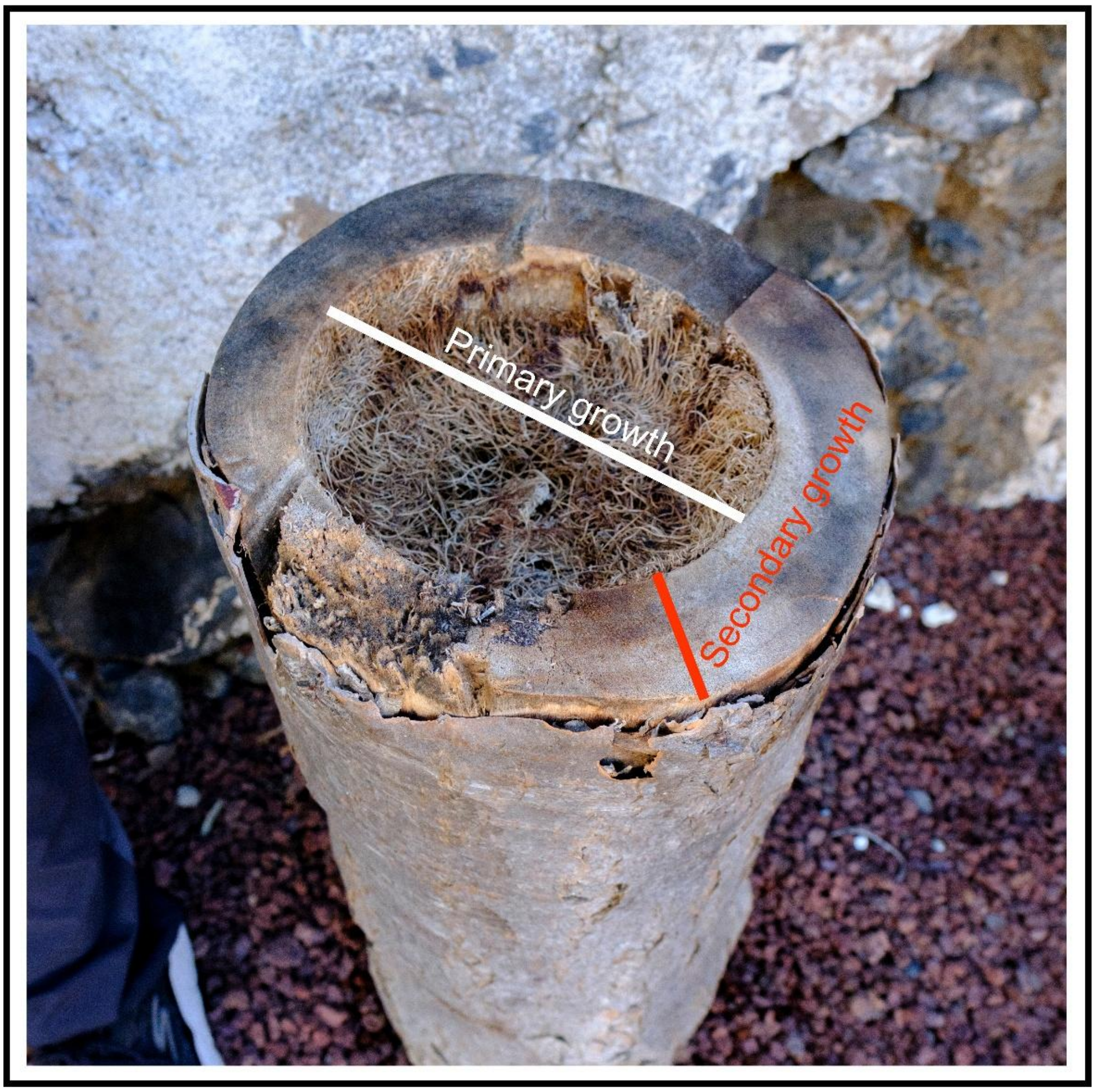

Figure S2. A *Dracaena draco* specimen (55% of canopy damage) with the multichannel resistivity system (PiCUS: TreeTronic, Argus Electronic GmbH) installed on the trunk. Electrodes can be seen connected to nails that were inserted 5–8 mm into the trunk, in contact with the tissues below the periderm.

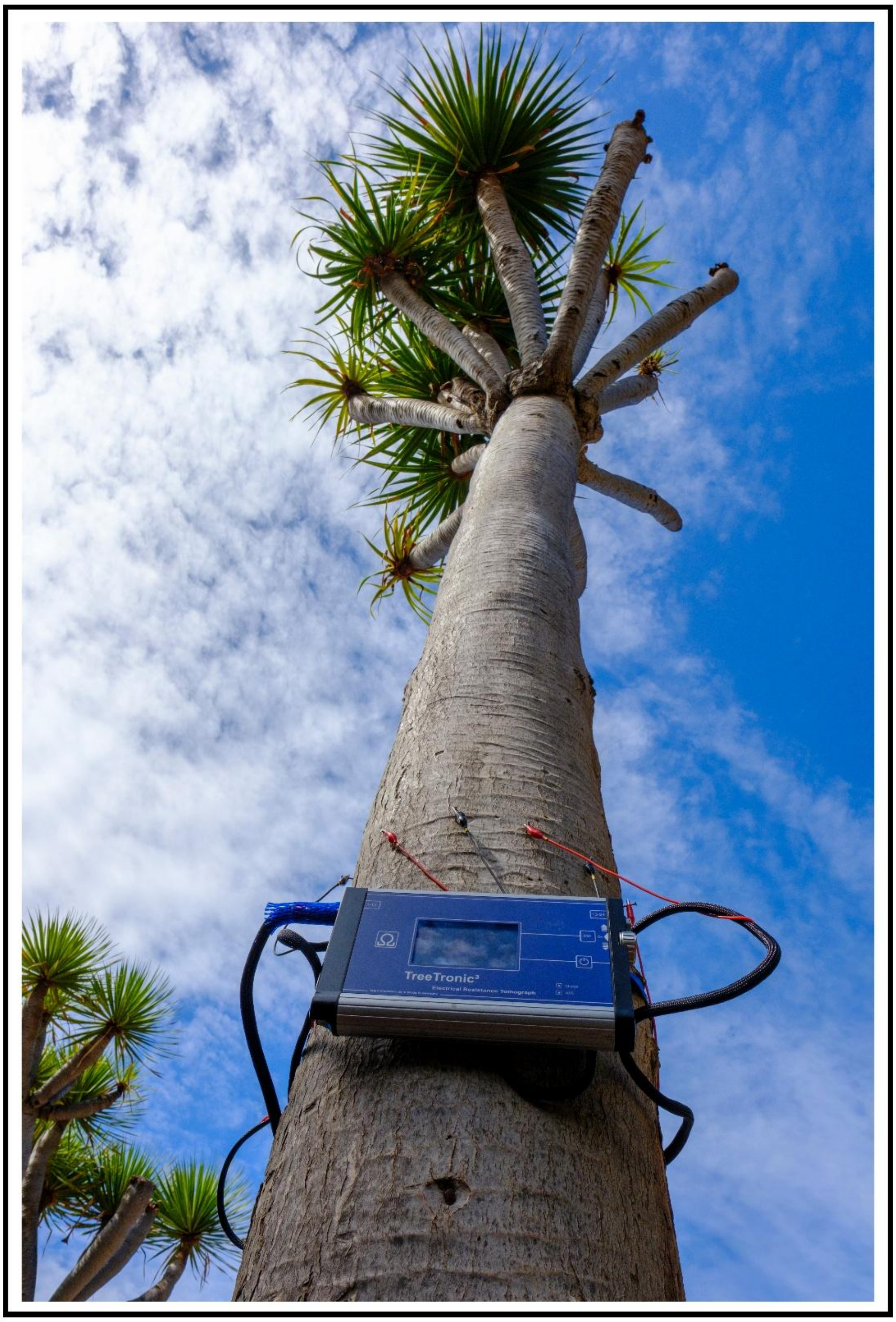

Figure S3. Electrical resistivity tomograms of three *Dracaena draco* trees per canopy damage level: visibly undamaged (with 0% damage; A, D and G), moderately damaged (25-55% of damage; C, E and H) and severely damaged (70-95% of damage; C, F and I) (see also Figure 1). The corresponding level of canopy damage is given on the right top of each tomogram. In tomograms, areas of low resistivity are indicated by blue color while areas of high resistivity are indicated by red color (note the tomogram-specific range of the scale).

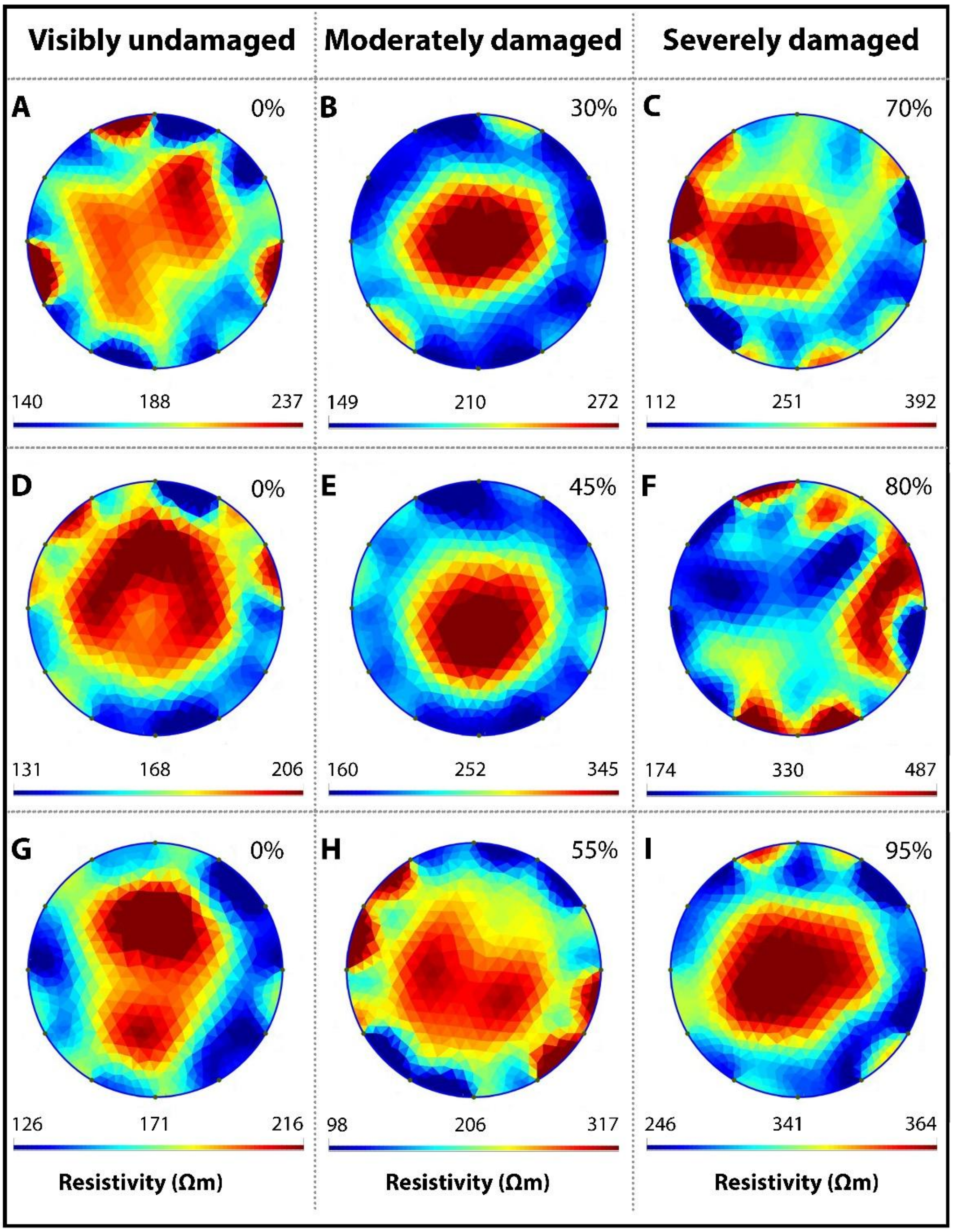

Figure S4. Tree height (cm) *versus* maximum (A), minimum (B), mean (C), central (D) and peripheral (E) electrical resistivity (Ωm) of *Dracaena draco* trunks of trees. Different colors and symbols indicate different damage classes: visibly undamaged (0%; green circles), moderately damaged (25-55%; yellow triangles) and severely damaged (70-95%; red squares) (see also Figure 1). The dashed line shows the linear regression, and the gray shaded area represents its 95% confidence interval.

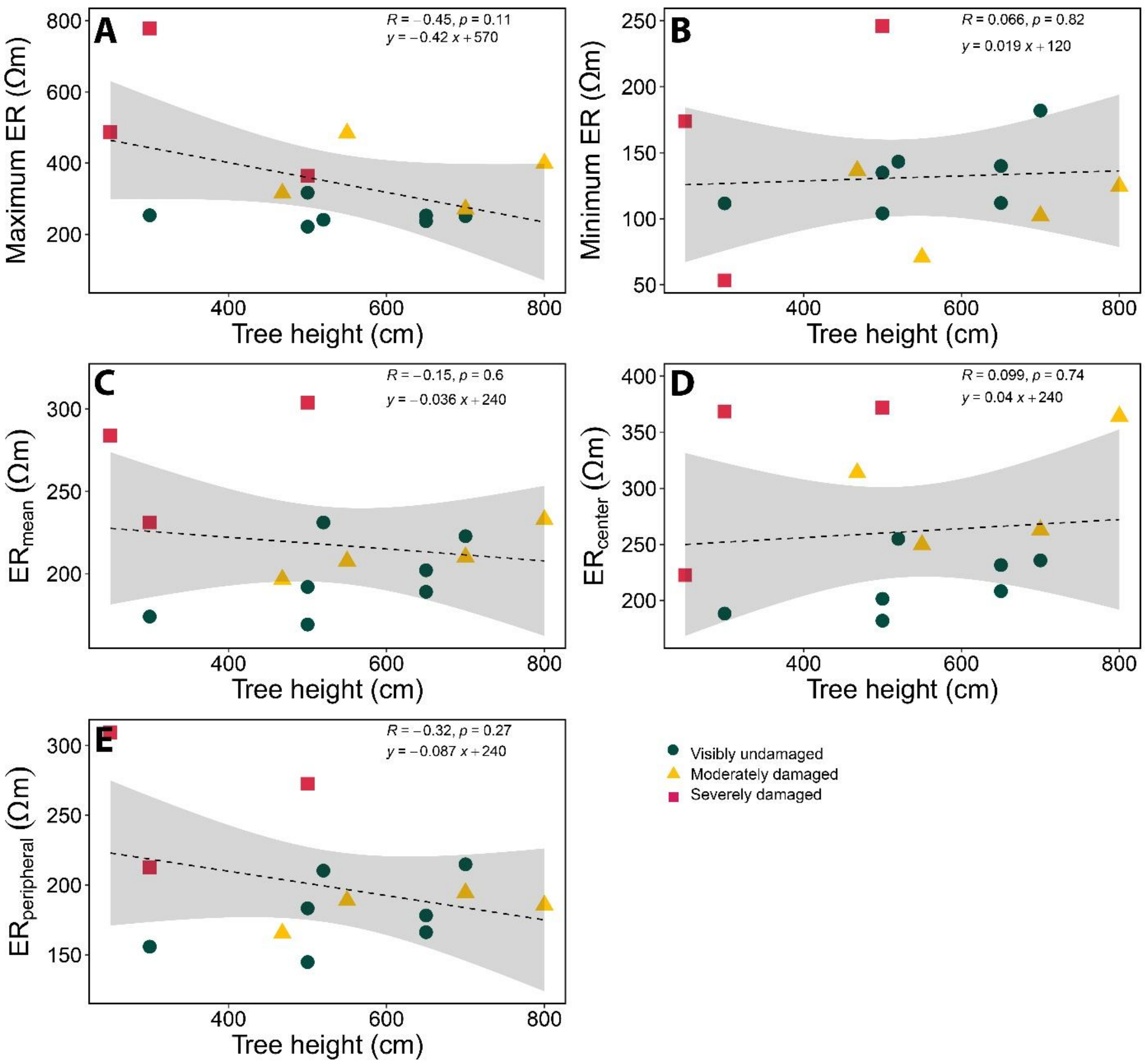

Supplement: Supplementary file 1 [file Image1.pdf]
